# Induction of protection in mice against a respiratory challenge by a vaccine formulated with exosomes isolated from *Chlamydia muridarum* infected cells

**DOI:** 10.1038/s41541-020-00235-x

**Published:** 2020-09-18

**Authors:** Sukumar Pal, Yeva Mirzakhanyan, Paul Gershon, Delia F. Tifrea, Luis M. de la Maza

**Affiliations:** 1 Department of Pathology and Laboratory Medicine, University of California, Irvine, Irvine, CA USA; 2 Department of Molecular Biology and Biochemistry, University of California, Irvine, Irvine, CA USA

**Keywords:** Infectious diseases, Vaccines, Vaccines, Protein vaccines

## Abstract

The goal of this study was to determine if exosomes, isolated from *Chlamydia muridarum* infected HeLa cells (*C. muridarum*-exosomes), induce protective immune responses in mice following vaccination using CpG plus Montanide as adjuvants. Exosomes, collected from uninfected HeLa cells and PBS, formulated with the same adjuvants, were used as negative controls. Mass spectrometry analyses identified 113 *C. muridarum* proteins in the *C. muridarum*-exosome preparation including the major outer membrane protein and the polymorphic membrane proteins. Vaccination with *C. muridarum*-exosomes elicited robust humoral and cell-mediated immune responses to *C. muridarum* elementary bodies. Following vaccination, mice were challenged intranasally with *C. muridarum*. Compared to the negative controls, mice immunized with *C. muridarum*-exosomes were significantly protected as measured by changes in body weight, lungs’ weight, and number of inclusion forming units recovered from lungs. This is the first report, of a vaccine formulated with *Chlamydia* exosomes, shown to elicit protection against a challenge.

## Introduction

*Chlamydia trachomatis* is the most common sexually transmitted bacterial pathogen in humans. According to the World Health Organization, ~130 million people worldwide are infected each year with *Chlamydia*^1^. In the USA, depending on the population studied, 5–20% of men and women become *C. trachomatis* positive during their reproductive years^2^. In countries with poor hygiene, *Chlamydia* can infect the eye causing trachoma^3^. About 75% of the genital infections in females and 50% in males are asymptomatic^4–6^. Genital infections in females can produce cervicitis, acute urethral syndrome, ectopic pregnancy, pelvic inflammatory disease (PID) and infertility while in males it may result in urethritis, epididymitis, prostatitis and testicular infarctions^2,6,7^. If a *Chlamydia* infection is not treated during pregnancy, 70% of newborns are infected^8^. Moreover, genital *C. trachomatis* infections are associated with other serious diseases such as cervical hypertrophy, induction of squamous metaplasia, and HIV and HPV infections^9,10^. Antibiotic therapy is available, but due to the high proportion of asymptomatic patients antibiotic treatment have failed to eradicate these infections^11,12^. Countries that established screening programs have observed an increase in the prevalence of genital infections likely due to the failure to develop natural immunity as a result of antibiotic therapy^13^. Therefore, *Chlamydia* infections remain a key public health priority and a vaccine is required to control them^14–20^.

A number of chlamydial antigens have been tested for their ability to confer protection against genital, respiratory or ocular challenges^14–20^. Of these, the *Chlamydia* major outer membrane protein (MOMP) is the most promising vaccine candidate^16,21–25^. A shortcoming of MOMP is that it induces serovar/serogroup-specific protection^23,26^. Thus, additional antigens may be required to induce broader protection^17,27^. Candidates, including a family of well-conserved polymorphic membrane proteins (Pmps) have been tested as vaccine antigens. Pmps have different relative expression levels in elementary bodies (EB) versus reticulate bodies (RB)^28^. Thus, it may be difficult to produce a vaccine that works against both forms. Furthermore; lower levels of protection were observed in Pmp-vaccinated versus MOMP-vaccinated mice^29^. Thus, there is a need to identify additional protective antigens.

Exosomes are 40–100 nm diameter membrane vesicles secreted by cells with potential roles in various physiological processes^30–32^. To date, very little information is available on the role(s) of exosomes secreted from *Chlamydia*-infected epithelial cells. Exosomes from other organisms have been shown to either protect against infection or lead to pathogenesis^33,34^. Here, we studied in mice the protective ability of *C. muridarum* exosomes against a respiratory challenge. Exosomes from *C. muridarum*-infected HeLa cells elicited robust humoral and cell-mediated immune responses and conferred significant protection against a respiratory challenge. Thus, antigens found in these exosomes can now be explored as potential vaccine candidates.

## Results

### Characterization of exosomes

Mass spectrometry analyses (MSA) thresholded at the 1% FDR level revealed a total of 113 *C. muridarum* proteins in *C. muridarum*-exosomes preparations (Supp. Table 1) comprising 12.3% of the 915-member *C. muridarum* proteome. In addition, MSA identified about six times more human proteins (1885) in *C. muridarum-*exosomes compared to the HeLa control exosomes in which only 331 human proteins were identified. In both types of exosomes 29 common protein contaminants were detected by MSA. Both types exosomes had exosomal protein markers including heat shock proteins, cytoskeletal proteins, and transcription and translation factors. Notable among the *Chlamydia* proteins present in the *C. muridarum* exosomes were: a family of Pmps (B-I), 60 kDa cysteine-rich protein, 42 kDa MOMP, 15 kDa cysteine-rich protein, four families of Chaperones (ClpB, Grow ES/EL, Dnak, Dnaj), virulence plasmid protein Pgp5, histone H1 like protein, protein translocase subunit SecA, various tRNA synthetases (listed as tRNA ligases), 30S and 50S ribosomal proteins, and eight uncharacterized *Chlamydia* proteins (TC-0248, TC-0268, TC-0311, TC-0561, TC-0708, TC-0713, TC-0825, TC-0873). As expected, no *Chlamydia* proteins were detected in control-exosomes preparations.

Nanoparticle tracking analyses estimated ~3.24 × 10^8^ ± 1.91 × 10^7^ exosomes/1 μg of protein in the *C. muridarum*-infected preparation and ~4.73 × 10^8^ ± 9.73 × 10^6^ particles/1 μg of protein in the non-infected control. The mean size of *C. muridarum*-exosomes was 106.1 ± 2.6 nm (mode 69.0 ± 4.6 nm) and the mean size of control-exosomes was 103.4 ± 2.6 nm (mode 70.0 ± 1.9 nm). Thus, in preparations collected from *C. muridarum* infected and non-infected HeLa cells the number and size of the exosomes were similar.

Label-free peptide quantitation (data not shown) allowed us to approximate the absolute abundance of each protein based on the three most abundant tryptic peptide species from each. On this basis, we could estimate total protein in the *chlamydia* exosomes preparation attributable to the chlamydia proteome to be just 6.27 mole%, with the remainder (93.73 mole%) attributable the human host (HeLa 229 cells). Among *Chlamydia* proteins only, 10.04 mole% corresponded to MOMP, while 81.72 mole% was from Pmps.

### Validation of chlamydial antigens in the exosomes

**Fig. 1:**
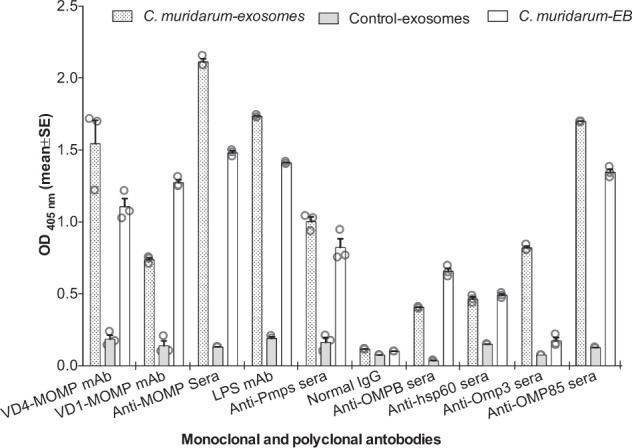
Reactivity of mAbs and polyclonal sera to *C. muridarum* antigens against *C. muridarum* exosomes. mAbs and polyclonal sera to *C. muridarum* antigens were used in an ELISA to identify proteins present in *C. muridarum* exosomes. *C. muridarum* EB were used as a positive control antigen.

To validate *C. muridarum* antigens present in the *C. muridarum-*exosomes, these were probed by an indirect ELISA with mAbs and mouse polyclonal sera raised against *Chlamydia* antigens. As shown in Fig. 1, exosomes contained LPS, MOMP, Pmps, putative outer membrane protein (OMP 85) and the 60 kDa cysteine-rich protein. No antibody bound with the control-exosomes. *C. muridarum*-exosomes not only bound to mAbs that recognize linear epitopes in VD1 and VD4 of *C. muridarum* MOMP but also with a mAb that binds to a conformational epitope present in the native MOMP trimer.

### Humoral immune responses following vaccination

To determine humoral immune responses, serum samples were collected from exosomes vaccinated mice the day before the i.n. challenge. Pre-immunization sera were used as controls. Antibody titers were determined using EB and MOMP (Table 1). Animals vaccinated with *C. muridarum*-exosomes had an IgG geometric mean titer (GMT) to EB of 25,600 (range: 25,600–25,600) while negative controls immunized with control-exosomes or PBS had titers below the detection level (<100). Animals vaccinated with *C. muridarum*-exosomes had an anti-MOMP IgG GMT of 9,051 (range: 6,400–12,800). No anti-MOMP or EB antibody titers were detected in sera of mice immunized with control-exosomes or PBS.

**Table 1:** Serum antibody titers of *C. muridarum*-exosomes vaccinated mice the day before the i.n. challenge.

Vaccine	GMT (range) to *C. muridarum* EB			GMT (range) to *C. muridarum* rMOMP			Serum neutralizing GMT (range)
	IgG	IgG1	IgG2a	IgG	IgG1	IgG2a	
*C. muridarum*-exosomes	25,600^a^ (25,600–25,600)	3200^a^ (1600–6400)	9051^a^ (6400–12,800)	9051^a^ (6400–12,800)	358^a^ (160–800)	9051^a^ (6400–12,800)	8611^a^ (5120–10,240)
Control-exosomes	<200	<200	<200	<100	<100	<100	<20
PBS	<200	<200	<200	<100	<100	<100	<20

^a^Significant (*P* < 0.05) by the Kruskal–Wallis test compared to the control-exosomes or PBS immunized groups

To determine whether the exosomes vaccine elicited Th1 or Th2-biased immune responses, the IgG2a/IgG1 ratios were calculated. Mice immunized with *C. muridarum*-exosomes had a ratio of 2.8 (9510:3200) against EB and a ratio of 25.3 (9051:358) to MOMP indicating robust Th1 biased immune responses (Table 1).

In vitro neutralizing antibody levels were determined in sera the day before the challenge (Table 1). Animals immunized with *C. muridarum*-exosomes had a neutralizing GMT of 8611 while mice vaccinated with control-exosomes or PBS had titers below the detection level (<50).

**Fig. 2:**
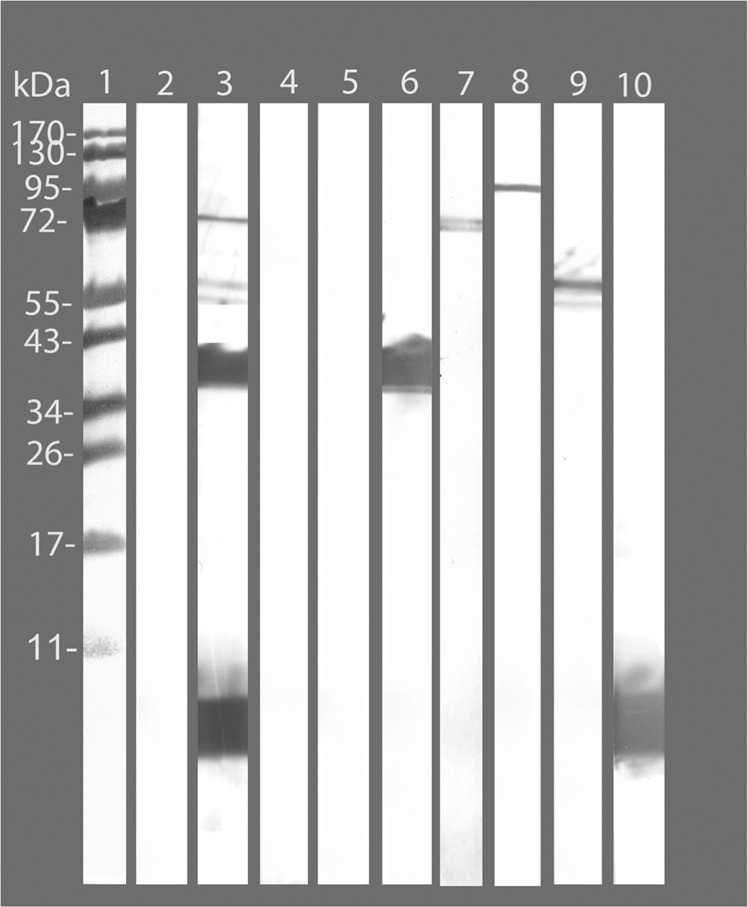
Western blot of serum samples from immunized mice. *C. muridarum* EBs were run on a 10% SDS-PAGE and blotted onto nitrocellulose paper. EBs were probed with pooled sera collected from *C. muridarum*-exosomes, control-exosomes, or PBS immunized mice. Lane 1, molecular weight standards. Sera from mice: Lane 2, pool pre-immune; Lane 3, d60 pool sera from *C. muridarum*-exosomes immunized mice; Lane 4, d60 pool sera from control-exosomes immunized mice; Lane 5, d60 pool sera from PBS immunized mice; Lane 6 mAb40 that recognizes the VD1 of *C. muridarum* MOMP; Lane 7, polyclonal sera from *C. muridarum* OMP85 immunized mice; Lane 8, pool sera from *C. trachomatis* serovar E Pmp (A-I) immunized mice; Lane 9, mAb02 to the *C. muridarum* 60 kDa cysteine-rich protein; Lane 10, mAb09 to *C. muridarum* LPS.

The western blot using sera collected the day before the i.n. challenge is shown in Fig. 2. Mice vaccinated with *C. muridarum*-exosomes had antibodies predominantly to bands of a high molecular weight protein (OMP 85), the 60 kDa cysteine rich protein, the 42 kDa MOMP, and LPS. Interestingly, no visible band corresponding to Pmps (≥100 kDa) was seen in the sera collected from *C. muridarum*-exosomes immunized mice. Control mice, immunized with control-exosomes or PBS, had no antibodies reactive with chlamydial components.

**Fig. 3:**
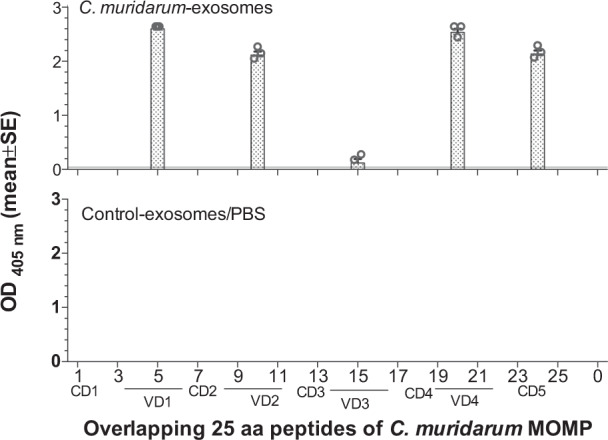
Binding of serum antibodies from mice immunized with *C. muridarum*-exosomes to synthetic *C. muridarum* MOMP peptides. Serum samples from mice immunized with *C. muridarum*-exosomes, control-exosomes, and PBS were collected the day before the intranasal challenge. Their reactivity to 25-aa overlapping peptides corresponding to the *C. muridarum* mature MOMP was analyzed by an ELISA.

To determine MOMP epitopes-specific antibodies, serum samples were probed with 25 aa overlapping *C. muridarum* MOMP peptides (Fig. 3). Antibodies from animals immunized with *C. muridarum*-exosomes recognized peptides located almost exclusively in the four variable domains (VD) and a constant domain (CD) 5 while no reactivity was obtained with sera from mice inoculated with control-exosomes or PBS.

### Cellular immune responses following vaccination

As a parameter of the *C. muridarum*-specific cellular immune responses, proliferation to *Chlamydia* antigens was determined using nylon wool purified spleen T-cells (Table 2). The delta counts per minute (Δ cpm: cpm from antigen-stimulated wells minus cpm from no antigen-stimulated wells) of T-cells from mice vaccinated with *C. muridarum*-exosomes EB was 14,462 ± 1899, while from animals inoculated with control-exosomes or PBS were 4730 ± 1392, and 779 ± 125, respectively (*P* < 0.05). The Δ cpm of all groups of mice stimulated with a positive stimulant, Con A, ranged from 65,987 to 78,567 indicating viability of the T cells. All negative control wells, that received only medium, had cpm that ranged from 637 to 2754.

**Table 2:** T-Cell-mediated immune responses of *C. muridarum*-exosomes vaccinated mice the day before the i.n. challenge.

Vaccine	Proliferative responses to (Δcpm ± 1SE		Medium mean cpm (±1SE)	IFN-γ responses (pg/ml ± 1SE)			IL-4 responses (pg/ml ± 1SE)		
	*Cm*-EB	Con A		*Cm* EB	Con A	Medium	*Cm* EB	Con A	Medium
*C. muridarum*-exosomes	14,462 ± 1899^a^	78,567 ± 8988	1163 ± 783	4789 ± 1379^a^	8089 ± 5629	<15	<4	100 ± 44	<4
Control-exosomes	4730 ± 1392	71,025 ± 8891	2754 ± 1117	<15	7,992 ± 1345	<15	<4	45 ± 10	<4
PBS	779 ± 125	65,987 ± 3825	637 ± 148	<15	5772 ± 1190	<15	<4	101 ± 9	<4

*cpm* counts per minute.

^a^Significant (*P* < 0.05) by the ANOVA compared to the control-exosomes or PBS immunized groups.

Mean IFN-γ levels (pg/ml), as a measure of a Th1 response, were determined in supernatants from EB-stimulated T-cells (Table 2). Animals immunized with *C. muridarum*-exosomes secreted the highest levels of IFN-γ (4789 ± 1379 pg/ml) compared to mice immunized with control-exosomes or PBS (<15 pg/ml). No IL-4 (<4 pg/ml; a Th2 marker) was detected in the T cells supernatants from animals immunized with either *C. muridarum*-exosomes, control-exosomes, or PBS following stimulation with *C. muridarum* EB. T cells stimulated with Con A secreted low levels of IL-4 in all immunized mice (45–101 pg/ml).

### Changes in body weight of mice following the i.n. challenge

**Fig. 4:**
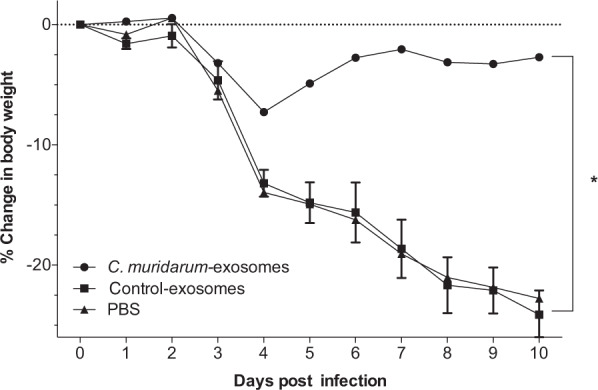
Daily changes in body weight following the i.n. challenge with *C. muridarum*. Mean percentage changes in daily body weight following the i.n. challenge with *C*. *muridarum* (*P < 0.05 by the Repeated Measures ANOVA).

As a measurement of the systemic effect of the infection, the body weight was determined for 10 days following the i.n. challenge. All mice lost weight for the first 2–4 days post-challenge (d.p.c.) (Fig. 4). Subsequently, mice vaccinated with *C. muridarum*-exosomes, slowly regained most of their initial body weight in contrast to mice immunized with control-exosomes or PBS. As determined by the repeated measures ANOVA, the cumulative body weight changes over the 10 days were significantly (*P* < 0.05) different between mice immunized with *C. muridarum*-exosomes and the two negative control groups. No significant differences were observed between the control-exosomes versus PBS group (*P* > 0.05).

**Fig. 5:**
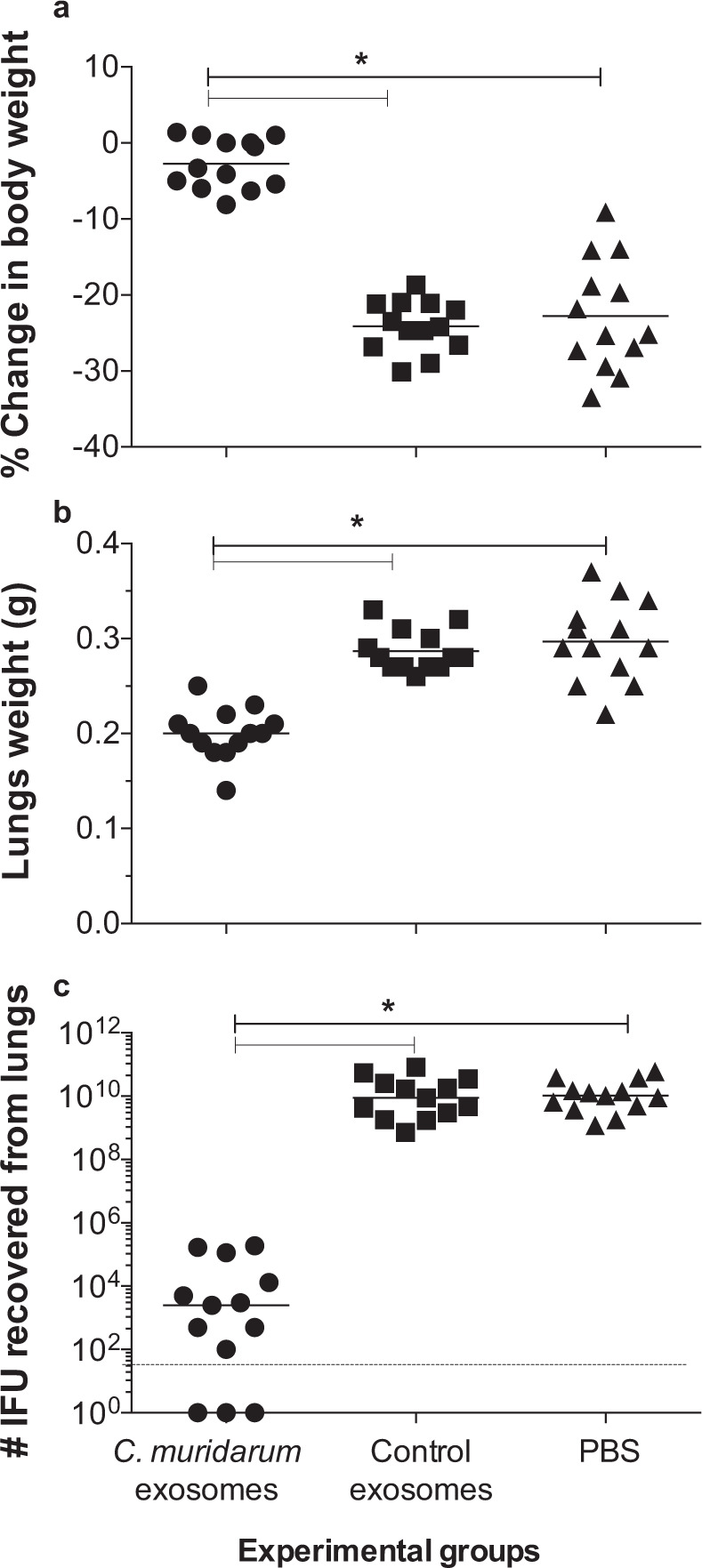
Disease burden at day 10 following the i.n. challenge with *C. muridarum*. **a** Percentage change in body weight at 10 days following the i.n. challenge. The mean is shown as a horizontal line. Each symbol represents an animal. Symbol “*” represents *P* value < 0.05 by the one-way ANOVA with Holm-Sidak multiple comparisons test. **b** Lungs weight (g) at 10 days after the i.n. challenge. The mean is shown as a horizontal line. Each symbol represents an animal. Symbol “*” represents *P* value < 0.05 by the one-way ANOVA with Holm-Sidak multiple comparisons test. **c** Number of *C. muridarum* IFU recovered from the lungs at day 10 after the i.n. challenge. The median is shown as a horizontal line. Each symbol represents an animal. Symbol “*” represents *P* value < 0.05 by the Kruskal–Wallis test with Dunn’s multiple comparisons.

By 10 d.p.c., mice immunized with *C. muridarum*-exosomes, had lost −2.7 ± 0.92% of their initial body weight (Fig. 5a). In contrast, at 10 d.p.c., mice vaccinated with PBS or control exosomes, had lost significantly more body weight compared to their initial body weight (−24.12 ± 0.93% and −27.8 ± 2.03%, respectively (*P* < 0.05).

### Lungs weight

As a parameter of local inflammatory responses, lungs weights were measured at 10 d.p.c. (Fig. 5b) The mean lungs weight from mice vaccinated with *C. muridarum*-exosomes (0.20 ± 0.01 g) was significantly lower than negative control mice immunized with exosomes or PBS (0.29 ± 0.01 g and 0.30 ± 0.01 g), respectively (*P* < 0.05).

### Burden of *C. muridarum* infection in the lungs

Ten days after the i.n. challenge, mice were euthanized and their lungs cultured for the detection of *C. muridarum* IFU (Fig. 5c). The median number of IFU recovered from lungs of mice vaccinated with *C. muridarum*-exosomes was 2.5 × 10^3^ (range: BLD–187 × 10^3^). In comparison, the mice vaccinated with either control-exosomes or PBS the median number of IFU recovered were 8.7 × 10^9^ (range: 0.7 × 10^9^–81 × 10^9^) and 10 × 10^9^ (range: 1.2 × 10^9^–59 × 10^9^), respectively (*P* < 0.05).

### Local immune responses in the lungs at 10 d.p.c

**Fig. 6:**
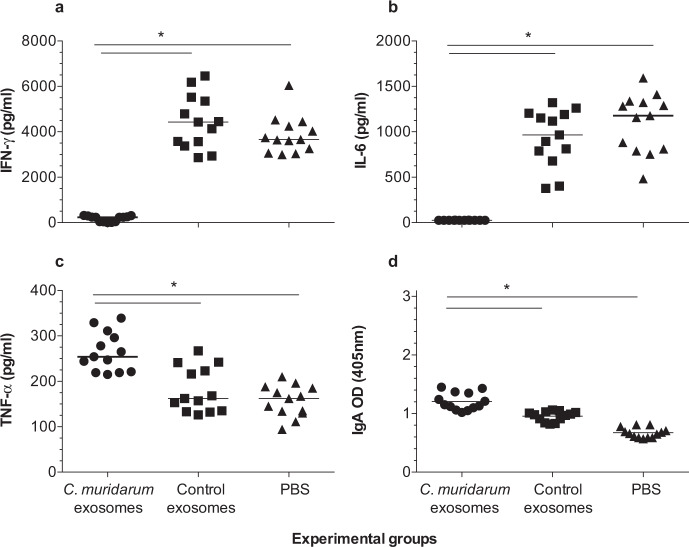
Local immunological responses in vaccinated mice at 10 days after challenge. **a** IFN-γ levels in the lungs at 10 days after challenge. The mean is shown as a horizontal line. Each symbol represents an animal. Symbol “*” represents P value less than 0.05 by the one-way ANOVA with Holm-Sidak multiple comparisons test. **b** IL-6 levels in the lungs at 10 days after challenge. The mean is shown as a horizontal line. Each symbol represents an animal. Symbol “*” represents *P* value < 0.05 by the one-way ANOVA with Holm-Sidak multiple comparisons test. **c** TNF-α levels in the lungs at 10 days after challenge. The mean is shown as a horizontal line. Each symbol represents an animal. Symbol “*” represents *P* value < 0.05 by the one-way ANOVA with Holm-Sidak multiple comparisons test. **d** *C. muridarum*-specific IgA levels in the lungs at 10 days after challenge. The mean is shown as a horizontal line. Each symbol represents an animal. Symbol “*” represents *P* value < 0.05 by the one-way ANOVA with Holm-Sidak multiple comparisons test.

To evaluate local immune responses, the lungs supernatants were collected at 10 d.p.c. and levels of IFN-γ, IL-6, TNF-α, and *C. muridarum*-specific IgA were determined (Fig. 6a–d). The mean levels of IFN-γ (pg/ml) in mice vaccinated with *C. muridarum*-exosomes was 175 ± 24 pg/ml indicating the control of *C. muridarum* infection in the lungs. Levels of IFN-γ were significantly higher in mice immunized with control exosomes (4434 ± 228), or PBS (3869 ± 162) (*P* < 0.05) suggesting the presence of active infection in the lungs (Fig. 6a). The levels of IL-6 follow a similar pattern to that observed for IFN-γ. The mean level of IL-6- (pg/ml) in mice vaccinated with *C. muridarum*-exosomes was 26 ±0 pg/ml indicating the clearance of inflammation in the lungs. Levels of IL-6 were significantly higher in mice immunized with control exosomes (936 ± 313), or PBS (1099 ± 323) (*P* < 0.05, Fig. 6b). In contrast, levels of TNF-α were significantly higher in mice immunized with *C. muridarum* exosomes (264 ± 43 pg/ml) compared to the control exosomes (181 ± 50), or PBS (156 ± 35) (*P* < 0.05, Fig. 6c).

As expected, the amounts of *C. muridarum*-specific IgA (OD_405_) followed an opposite trend compared to the levels of IFN-γ (Fig. 6d). Mice vaccinated with *C. muridarum*-exosomes had high levels of IgA (*A*_405_ 1.21 ± 0.04) compared to the animals immunized with control-exosomes (*A*_405_ 0.96 ± 0.02) or PBS (*A*_405_ 0.67 ± 0.02) (*P* < 0.05).

## Discussion

This study aimed to determine the ability of exosomes, collected from *C. muridarum* infected HeLa cells, to protect against an intranasal challenge with *C. muridarum*. MSA of exosomes from *C. muridarum*-infected cells identified 113 *C. muridarum* proteins including some previously described protective antigens, such as MOMP and Pmps, and others not previously tested in vaccines^22,29,35^. NTA determined similar number and size exosomes from the *C. muridarum*-infected and the non-infected HeLa preparations indicating they are membrane vesicles. Vaccination with exosomes from *C. muridarum* infected HeLa cells adjuvanted with CpG and Montanide elicited preferentially Th1 type humoral and cell-mediated immune responses and produced high levels of *C. muridarum-*specific neutralizing antibodies in serum. Protection against a respiratory challenge with *C. muridarum* was determined by the changes in body weight during the 10 days post-infection, lungs weight and number of IFU recovered from the lungs at day 10 post-infection. To our knowledge, this is the first time that exosomes from *C. muridarum* infected cells have been shown to elicit protection against a chlamydial challenge. The novel discovery of *Chlamydia* exosomes as a source of protective antigen(s) should be further explored.

A large volume of literature had linked exosomes with the pathophysiology of many diseases^31,36^. In addition, the protective role of exosomes against bacterial or parasitic infection is emerging^33,37,38^. For example, vaccination with outer membrane vesicles (OMV) produced by Δ*tol*R deleted *Salmonella* strains was shown to lower *Salmonella* burden in mice^39^. Similarly, a vaccination study with exosomes collected from *Plasmodium yoelli* infected reticulocytes was shown to protect mice against a lethal *P. yoelli* challenge^33^. Mice, immunized with extracellular vesicles collected from the adult gastrointestinal nematode *Heligmosomoides polygyrus*, developed protective immunity against a larval challenge^38^. Furthermore, there is an effective OMV–based human vaccine against *Neisseria meningitidis* already in the market^40^. These studies, along with this one, support the protective role of exosomes against some infections.

MSA of *C. muridarum* exosomes, revealed several important findings. Of the proteins identified in the exosomes preparation, label free peptide quantitation, detected only 6.7 mole% of total protein attributed to *Chlamydia* and the remainder (93.3 mole%) to human host cells. Moreover, exosomes from *C. muridarum*-infected HeLa cells had 6-fold greater diversity of human proteins than control exosomes collected from uninfected-HeLa cells. This suggests that *Chlamydia* infection may lead to an increase in the number of host cells proteins in the exosomes. This may result in an increase in the quantity or quality of signals sent via exosomes to host cells. More work needs to be done to define the chlamydia-host cell interface at the exosomal level.

Among the 113 *Chlamydia* proteins identified by MSA, ~10 mole% was attributable to MOMP while 82 mole% was assigned to Pmps. Importantly, these amounts were sufficient to elicit significant protective immunity against the pulmonary challenge suggesting high potencies of these antigens. Considering that exosomes elicited MOMP specific immune responses we have to assume that they played a role in protection. Also, T cells epitopes in Pmps^41,42^ might have helped vaccinated mice to develop T cell-mediated immunity against *C. muridarum*. By western blot, we could not detect Pmp reacting antibodies. Antibody responses to Pmps in humans are variable and even gender specific. Hence Pmps are considered to be mainly T cell antigens^42^.

Of the 113 *C. muridarum* proteins identified by MSA, eight (TC-0248, TC-0268, TC-0311, TC-0561, TC-0708, TC-0713, TC-0825, and TC-0873), are uncharacterized and have not been tested for their ability to induce protection. Thus, it will be important to analyze their potential protective function. Russell et al.^43^, using MSA, identified 32 proteins in *C. muridarum* exosomes collected from infected mouse oviduct epithelial cells. Although there are common proteins in both experiments, more proteins were identified in this study^43^. One noticeable difference is the absence of MOMP in Russel et al. publication^43^. However, another study by Frohlich et al.^44^ on microvesicles, isolated from *C. trachomatis*-infected cells, supports our MOMP findings. The difference in the exosomes MSA profiles may be due to the cell lines used (HeLa 229, L929, or mouse oviduct epithelial cells), culture conditions, strains of *Chlamydia* utilized to infect cells, and methods chosen for collection and analyses.

Exosomes, due to their intrinsic adjuvant capabilities, may have modulated *Chlamydia* antigen presentation in antigen-presenting cells. Russell et al.^43^ demonstrated that exosomes, collected from *Chlamydia*-infected cells, were capable of maturing dendritic cells and inducing high levels of Th1/Th2 promoting cytokines such as, IL-6, IL-10, IL-12, IFN-γ and TNF-α and chemokines especially keratinocyte chemoattractant (CXCL-1), MCP-1 (CCL2), MIP-1α (CCL3), Rantes (CCL5), and Eotaxin (CCL1).

Drawbacks of using exosomes as antigens, are the presence of host cell components and chlamydial heat shock proteins (hsp) (96, 70, 60, and 40 kDa). Antibodies to *Chlamydia* 60 kDa hsp have been associated with the development of long-term pathogenesis such as PID, infertility, and trachoma^45^. Thus, it is highly possible that chlamydial exosomes containing 60 kDa hsp may be involved with the development of long-term pathogenesis. In vivo studies are needed to delineate the role of exosomes in the pathogenesis of chlamydial infections.

One of the limitations of this study is that the role of mRNA or miRNA in eliciting protective immunity in mice was not evaluated. In addition to protein antigens, exosomes-associated mRNAs could potentially translate proteins in the target/antigen-presenting cells. Similarly, miRNA could modulate gene expression of immune cells^46,47^. Thus, studies are needed to define the role of mRNA and miRNA in exosomes. Also, the limited yield of exosomes from infected cells may discourage mass scale production for vaccine studies. This limitation of low yield may be overcome by producing recombinant OMV. Indeed, a recombinant OMV carrying *C. muridarum* serine protease HtrA has been shown to elicit *C. muridarum*-specific neutralizing antibodies in mice^48^. Further studies are needed to determine if recombinant OMV carrying MOMP, Pmps or some of the novel antigens found here, can produce protective immunity.

In conclusion, we have shown for the first time that exosomes from *Chlamydia* infected cells can induce protective immunity in mice against a respiratory challenge. These new *Chlamydia* antigens present in exosomes should be tested to determine their ability to elicit protective immune responses.

## Methods

### Stocks of *C. muridarum*

*C. muridarum* strain NiggII was grown in HeLa-229 cells using Eagle’s minimal essential medium (MEM) supplemented with 5% fetal bovine serum (FBS). To produce antigens for ELISA and western blot *C. muridarum* was also grown in McCoy cells. EB were purified as described and stored in sugar phosphate glutamate buffer (SPG) at −80 °C^49^. The number of *C. muridarum* inclusion forming units (IFU) was determined in HeLa-229 cells by an immunoperoxidase staining with *C. muridarum*-specific mAbs^21^.

### Preparation of exosomes

Exosomes from *C. muridarum* infected HeLa-229 cells were prepared as described^50^. HeLa-229 cells, grown in T175 flasks, were infected with *C. muridarum* (MOI 1–3) and were cultured in serum free Dulbecco’s modified Eagle medium containing cycloheximide (1 μg/ml). At 30 hours post-infection the media was collected and centrifuged at 16,500 × *g* for 1 h. The supernatants were passed through 0.22 μm membrane filters to remove EB and were centrifuged at 120,000 × *g* for 70 min. The pellets were collected, washed twice with PBS, resuspended in PBS, and stored at −80 °C. Control-exosomes were produced using uninfected HeLa-229 cells. The protein content in exosomes was measured by a BCA assay (Pierce, Rockford, IL).

### Mass spectrometry analyses of exosomes

Twenty μg of *C. muridarum*- or control-exosomes were denatured in 8 M urea/0.1 M triethylammonium bicarbonate buffer (TEAB)/10 mM tris(2-carboxyethyl)phosphine, pH 8.0, for 30 min at 37 °C with occasional cuphorn ultrasonication. Samples were then diluted to 6 M urea with 0.1 M TEAB, pH 8.0, and incubated overnight with LysC (Promega, Madison, WI) at 1:100 enzyme:protein ratio, before diluting to 1 M urea with 0.1 M TEAB, pH 8.0, and incubation overnight with Trypsin Gold (Promega) at a 1:100 protein:enzyme ratio. The resulting samples were each acidified with formic acid (FA) to 2% FA final and desalted using a C18/SCX stagetip^51^. Peptides were eluted from stagetips with 5% NH_4_OH / 80% CH_3_CN then dried under vacuum and redissolved in 0.1% FA in water for mass spectrometry analyses (MSA; nanoLC-MS/MS).

Using an Easy-nLC 1000, a portion of each sample was injected to a 250 × 0.075 mm (ID) nanocapillary column packed in-house with C18 ReproSil-Pur (1.9 micron), eluting the column with a gradient of CH_3_CN in 0.1% FA (0–5% over 5 min extending to 25% over 205 min and to 35% CH_3_CN over a further 30 min) at a flow rate of 250 nano liter/min. The column eluate was sprayed into an LTQ Orbitrap Velos Pro mass spectrometer, collecting precursor spectra in the range 380–1600 m/z. Up to 15 of the most intense ions in each precursor spectrum with a charge of +2 to +4 and a minimum signal of 5000 were fragmented by HCD with normalized collision energy of 30%. Ions were dynamically excluded for 40 s after two fragmentations within 30 s, via a 500-entry list, with early expiration from the list after a detection within the exclusion period falling below S/N = 2.0.

### MSA search and quantitation

Raw file data were processed to peaklists by Mascot Distiller 2.7.1. Using Mascot 2.6.1, each resulting mass list was subjected to target-decoy searching against SwissProt (02/2019) plus a library of common contaminants (taxonomy: Human; *Chlamydia muridarum*), with Trypsin enzyme specificity, a maximum of 1 missed cleavage, parent and product mass tolerances of 20 ppm, and variable modifications of Deamidated (NQ) and Oxidation (M). Results were thresholded at *p* = 0.05 yielding a corresponding FDR of <5%. In-house software was used to generate a matrix of protein accessions (rows) vs. samples (columns). Using Mascot Distiller 2.7.1, data were re-searched as above, and the resulting peptides subjected to label-free quantitation with protein abundances scored from the summed intensities of the top 3 most intense peptides per protein and the resulting values ratioed for infected:uninfected samples. Only the highest scoring protein from each protein family was retained. Ratios were sorted in descending order and charted using Microsoft Excel.

### Characterization of exosomes by nanoparticle tracking analyses (NTA)

Purified exosomes collected from *C. muridarum* infected-HeLa cells and from control non-infected HeLa cells were diluted to 1 μg/ml in 10 mM PBS, pH 7.2, for size characterization by NTA using a NanoSight 300 (Malvern Panalytical, UK). Each particle in a field of view was tracked by detecting scattered light at 488 nm and was analyzed by a proprietary software NTA (Malvern Panalytical, UK). Three videos were recorded (60 s each). All experiments were performed at room temperature.

### Immunization, challenge, and data collection

Three-week old BALB/c (H-2^d^) mice were immunized with *C. muridarum*- or control-exosomes, (10 μg/mouse/immunization), three times at two-week intervals by the intramuscular (i.m.) plus the subcutaneous (s.c.) routes. CpG-1826 (10 µg/mouse/immunization) and Montanide ISA 720 VG (70:30 v/v) were used as adjuvants^21^. Another negative control group was vaccinated with PBS adjuvanted as above following the same schedule.

Four weeks after the final immunization, anesthetized mice were challenged i.n. with 10^4^ IFU of *C. muridarum* and mice were weighed daily for 10 days^22,52^. At 10 days post-challenge (d.p.c.) mice were euthanized, their lungs weighed and homogenized in SPG. Serial dilutions of the homogenates were used to infect Hela-229 cells grown in 48-well plates. Cells were then incubated for 30 h at 37 °C in a 5% CO_2_ incubator. Inclusions were visualized with *Chlamydia*-specific monoclonal antibodies (mAbs) and counted^53^. The limit of detection (BLD) was <50 *C. muridarum* IFU/lungs/mouse. We had complied with all relevant ethical regulations for animal testing and research. Animal protocols were approved by the University of California Irvine, Animal Care and Use Committee.

### Immunological assays

Blood from the periorbital plexus was collected before immunization and the day before the challenge^27^. To avoid the cross-reactivity between the HeLa cells proteins present in the EB preparation and the antibodies that might have developed in mice vaccinated with exosomes collected from HeLa cells, EB grown in McCoy cells were used as antigen. *C. muridarum* EB produced in McCoy cells (10 μg/ml) or recombinant *C. muridarum* MOMP (1 μg/ml) were used as antigens. Goat anti-mouse IgG, IgG1, and IgG2a (BD Bioscience, San Diego, CA) diluted 1:5000 for IgG and 1:1,000 for the two isotypes, were used to determine the sub-class or isotype-specificity. ABTS [2, 2′-azino-bis-(3-ethylbenzthiazoline-6-sulfonate)] (Sigma-Aldrich, St. Louis, MO) was used as the substrate and the, plates were scanned in an ELISA reader at 405 nm (Labsystem Multiscan; Helsinki, Finland).

In vitro neutralization assays were performed according to Peterson et al. with modifications^54^. In brief, duplicate sets of two-fold serial dilutions of serum were made in Ca^+2^/Mg^+2^ free PBS containing 5% guinea pig serum as a source of complement. The serum samples were incubated with 10^4^ *C. muridarum* IFU for 45 min at 37 °C. The mixture was used to inoculate HeLa-229 monolayers grown in flat bottom 96-well plates which were centrifuged for 1 h at 1000 × *g*. The cells were incubated for 30 h. in culture medium with cycloheximide (1 µg/ml). Monolayers were then fixed and chlamydial IFU were stained with a *C. muridarum*-specific mAb 40 recognizing the *C. muridarum* MOMP^54^. The number of IFU was counted and neutralization was defined as greater than, or equal to, a 50% decrease in the number of IFU with respect to the controls incubated with pre-immunization sera^21^.

A T-cell lymphoproliferative assay (LPA) was performed using splenocytes collected from the immunized mice on the day immediately prior to challenge^53^. Splenic T-cells, purified using nylon wool (>95% purity), were stimulated with *C. muridarum* EB in the presence of antigen-presenting cells (APC). APC were pre-prepared by irradiation (3300 rads, ^137^Cs) of syngeneic splenocytes and 1.25 × 10^5^ cells were incubated in round bottom 96-well plates (Costar, Corning Inc.) at 37 °C for 2 h with EB at a 1:1 ratio. T cells were added to APC at a ratio of 1:1. Concanavalin A (5 μg/ml) served as a positive stimulant and cell culture medium (RPMI with 10% FBS) used as a negative antigen control. Following 96 h of incubation, cell proliferation was measured by adding 1 µCi of [methyl ^3^H] thymidine per well. The mean counts per minute (CPM) were determined from triplicate culture wells using a scintillation counter.

Levels of IFN-γ, TNF−α, IL-4, and IL-6 in stimulated T-cell supernatants, or in lung homogenates, were determined using commercial kits (BD Pharmingen, San Diego, CA)^24,27^.

Western blot analysis of sera from exosomes-immunized mice was conducted as described before^52^. Briefly, ~200 μg of *C. muridarum* EB were solubilized in SDS then separated on a 7.5 cm wide Tricine-sodium dodecyl sulfate–polyacrylamide slab gel. Serum samples were diluted 1:100 and added to the membrane strips in BLOTTO followed by overnight incubation at 4 °C. Detection of antibody binding employed horseradish peroxidase-conjugated goat anti-mouse antibody, and the signal was developed with 0.01% H_2_O_2_ and 4 chloro-1-naphthol. mAbs recognizing MOMP, the 60 kDa cysteine rich protein, and LPS and polyclonal sera from Pmp or OMP85 immunized mice were used as positive controls. All blots were derived from the same experiment and processed in parallel.

To detect antibodies, elicited by exosomes vaccination, to linear epitopes of *C. muridarum* MOMP, overlapping 25-mers corresponding to the amino acid sequence of mature MOMP, were chemically synthesized (SynBioSci Corp.; Livermore, CA)^24^. Peptide 25 (p25) overlapped the N- and C-termini of MOMP. The peptides were adsorbed onto high binding affinity ELISA plates (1 µg/well of a 96-well plate) and antibody binding was determined in triplicates as described above using anti-mouse IgG^55^.

### Validation of *Chlamydia* antigens in exosomes

To validate *Chlamydia* antigens, 100 μl of exosomes solution (5 μg/ml) were coated in triplicate overnight at 4 °C in 96-well plates, in 0.05 M carbonate bicarbonate buffer pH 9.6. The reactivity of the antigens was tested by ELISA, using mAbs or polyclonal mouse sera recognizing various *Chlamydia* antigens^52^. Goat anti-mouse Pan-Ig (KPL) diluted 1:1000 was added as secondary antibody. The signal was developed using ABTS [2, 2′-azino-bis-(3-ethylbenzthiazoline-6-sulfonate)] (Sigma-Aldrich, St. Louis, MO) and the plates were scanned in an ELISA plate reader at 405 nm (Labsystem Multiscan; Helsinki, Finland).

### Statistical analyses

The Kruskal-Wallis test with Dunn’s multiple comparisons was used to compare the numbers of *C. muridarum* IFU. The one-way ANOVA with Holm-Sidak multiple comparisons test was used to compare T-cell proliferative responses, levels of cytokines, lungs, and body weight changes of mice. Repeated measures ANOVA was employed to compare changes in mean body weight over the 10 days of observation.

### Reporting summary

Further information on research design is available in the Nature Research Reporting Summary linked to this article.

## Supplementary information

Supplementary Table

Reporting Summary

## Data Availability

The authors confirm that all relevant data are included in the paper and its Supplementary Information.
