# Supplementary material for: Induction of protection in mice against a respiratory challenge by a vaccine formulated with exosomes isolated from Chlamydia muridarum infected cells
Source: NPJ Vaccines. 2020 Sep 18;5:87. doi: 10.1038/s41541-020-00235-x (PMC7501220; doi:10.1038/s41541-020-00235-x)
Supplement: Supplementary file 1 — Supplementary Table [file 41541_2020_235_MOESM1_ESM.pdf]

Supplementary Table1: *C. muridarum* proteins identified in exosomes by mass spectrometry

Accession	Description	Mascot protein score
PMPD_CHLMU	Probable outer membrane protein PmpD	6046
PMPI_CHLMU	Probable outer membrane protein PmpI	5891
PMPG_CHLMU	Probable outer membrane protein PmpG	1702
EFG_CHLMU	Elongation factor G	1092
PMPB_CHLMU	Probable outer membrane protein PmpB	975
MOMPM_CHLMU	Major outer membrane protein	941
PMPF_CHLMU	Probable outer membrane protein PmpF	905
OMCB_CHLMU	Large cysteine-rich periplasmic protein OmcB	823
RPOC_CHLMU	DNA-directed RNA polymerase subunit beta	789
PMPE_CHLMU	Probable outer membrane protein PmpE	556
EFTU_CHLMU	Elongation factor Tu	535
Y248_CHLMU	Uncharacterized protein TC_0248	534
TOLB_CHLMU	Protein TolB homolog	439
PMPH_CHLMU	Probable outer membrane protein PmpH	421
RPOA_CHLMU	DNA-directed RNA polymerase subunit alpha	364
Y268_CHLMU	Uncharacterized protein TC_0268	330
RL4_CHLMU	50S ribosomal protein L4	329
OMP2_CHLMU	Outer membrane protein B	288
TIG_CHLMU	Trigger factor	273
CH60_CHLMU	60 kDa chaperonin	270
DEGPL_CHLMU	Probable periplasmic serine endoprotease DegP-like	266
TAL_CHLMU	Transaldolase	260
CLPX_CHLMU	ATP-dependent Clp protease ATP-binding subunit ClpX	234
RPOB_CHLMU	DNA-directed RNA polymerase subunit beta	229
GLGA_CHLMU	Glycogen synthase	220
KAD_CHLMU	Adenylate kinase	211
RRF_CHLMU	Ribosome-recycling factor	204
Y311_CHLMU	Uncharacterized protein TC_0311	192
RIR1_CHLMU	Ribonucleoside-diphosphate reductase subunit alpha	189
SYW_CHLMU	Tryptophan--tRNA ligase	183
SYY_CHLMU	Tyrosine--tRNA ligase	183
PYRH_CHLMU	Uridylate kinase [56]	181
RS2_CHLMU	30S ribosomal protein S2	177
SKPL_CHLMU	Skp-like protein	172
DNAK_CHLMU	Chaperone protein DnaK	165
RL25_CHLMU	50S ribosomal protein L25	142
Y873_CHLMU	Uncharacterized protein TC_0873	137
DCD_CHLMU	dCTP deaminase	137
DNAJ_CHLMU	Chaperone protein DnaJ	135
GLYA_CHLMU	Serine hydroxymethyltransferase	133
SRP_CHLMU	Sulfur-rich protein	125
RS7_CHLMU	30S ribosomal protein S7	122
RS5_CHLMU	30S ribosomal protein S5	122
SYA_CHLMU	Alanine--tRNA ligase	113
GREA_CHLMU	Transcription elongation factor GreA	111
RL2_CHLMU	50S ribosomal protein L2	111
CLPB_CHLMU	Chaperone protein ClpB	107
SURE_CHLMU	5~-nucleotidase SurE	106

MIP_CHLMU	Peptidyl-prolyl cis-trans isomerase Mip	105
RS9_CHLMU	30S ribosomal protein S9	103
CLPP1_CHLMU	ATP-dependent Clp protease proteolytic subunit 1	103
CLPC_CHLMU	Probable ATP-dependent Clp protease ATP-binding subunit	98
Y921_CHLMU	UPF0159 protein TC_0921	98
GPDA_CHLMU	Glycerol-3-phosphate dehydrogenase [NAD(P)+]	97
GYRA_CHLMU	DNA gyrase subunit A	95
DPO3B_CHLMU	Beta sliding clamp	94
IF2_CHLMU	Translation initiation factor IF-2	92
RS3_CHLMU	30S ribosomal protein S3	89
RS8_CHLMU	30S ribosomal protein S8	87
SYE_CHLMU	Glutamate--tRNA ligase	86
RL19_CHLMU	50S ribosomal protein L19	84
RS11_CHLMU	30S ribosomal protein S11	83
HCT1_CHLMU	Histone H1-like protein HC1	82
AAXA_CHLMU	Porin AaxA	82
RL14_CHLMU	50S ribosomal protein L14	81
GP5D_CHLMU	Virulence plasmid ParA family protein pGP5-D	77
PMPC_CHLMU	Probable outer membrane protein PmpC	75
Y344_CHLMU	Putative zinc metalloprotease TC_0344	70
SUCC_CHLMU	Succinate--CoA ligase [ADP-forming] subunit beta	66
TLC1_CHLMU	ADP,ATP carrier protein 1	66
GATA_CHLMU	Glutamyl-tRNA(Gln) amidotransferase subunit A	65
RS14_CHLMU	30S ribosomal protein S14	64
Y825_CHLMU	Uncharacterized protein TC_0825	62
RS12_CHLMU	30S ribosomal protein S12	62
PQQCL_CHLMU	PqqC-like protein	61
FABG_CHLMU	3-oxoacyl-[acyl-carrier-protein] reductase FabG	59
6PGD_CHLMU	6-phosphogluconate dehydrogenase, decarboxylating	55
RS6_CHLMU	30S ribosomal protein S6	54
RSMA_CHLMU	Ribosomal RNA small subunit methyltransferase A	54
GYRB_CHLMU	DNA gyrase subunit B	54
RS16_CHLMU	30S ribosomal protein S16	53
GPMA_CHLMU	2,3-bisphosphoglycerate-dependent phosphoglycerate mutase	53
RL17_CHLMU	50S ribosomal protein L17	51
PYRG_CHLMU	CTP synthase	51
RL1_CHLMU	50S ribosomal protein L1	50
SIGA_CHLMU	RNA polymerase sigma factor SigA	49
SODM_CHLMU	Superoxide dismutase [Mn]	49
RNR_CHLMU	Ribonuclease R	48
MAP1_CHLMU	Methionine aminopeptidase	48
RECA_CHLMU	Protein RecA	47
RL6_CHLMU	50S ribosomal protein L6	46
RL15_CHLMU	50S ribosomal protein L15	44
G6PD_CHLMU	Glucose-6-phosphate 1-dehydrogenase	43
TRXB_CHLMU	Thioredoxin reductase	43
PGK_CHLMU	Phosphoglycerate kinase	42
Y713_CHLMU	UPF0158 protein TC_0713	41
RL21_CHLMU	50S ribosomal protein L21	41
Y708_CHLMU	Uncharacterized protein TC_0708	40
FABH_CHLMU	3-oxoacyl-[acyl-carrier-protein] synthase 3	40
RL13_CHLMU	50S ribosomal protein L13	37

RS18_CHLMU	30S ribosomal protein S18	36
RL16_CHLMU	50S ribosomal protein L16	36
FOLD_CHLMU	Bifunctional protein FolD	35
RL24_CHLMU	50S ribosomal protein L24	34
GATB_CHLMU	Aspartyl/glutamyl-tRNA(Asn/Gln) amidotransferase subunit B	34
SECA_CHLMU	Protein translocase subunit SecA	34
NTPP_CHLMU	Nucleoside triphosphate pyrophosphatase	34
DER_CHLMU	GTPase Der	32
Y561_CHLMU	Uncharacterized protein TC_0561	26
CLPP2_CHLMU	ATP-dependent Clp protease proteolytic subunit 2	24
NUSG_CHLMU	Transcription termination/antitermination protein NusG	24
SYDND_CHLMU	Aspartate--tRNA(Asp/Asn) ligase	23
RL5_CHLMU	50S ribosomal protein L5	22
